# Investigation of Security Threat Datasets for Intra- and Inter-Vehicular Environments

**DOI:** 10.3390/s24113431

**Published:** 2024-05-26

**Authors:** Achref Haddaji, Samiha Ayed, Lamia Chaari Fourati, Leila Merghem Boulahia

**Affiliations:** 1 LIST3N, University of Technology of Troyes, 10300 Troyes, France; haddajiachref7@gmail.com (A.H.); samiha.ayed@utt.fr (S.A.); 2 National School of Electronics and Telecommunications of Sfax, Sfax 3052, Tunisia; 3 Digital Research Center of Sfax (CRNS), Laboratory of Signals, Systems, Artificial Intelligence, Networks (SM@RTS), Sfax University, Sfax 3029, Tunisia; lamiachaari1@gmail.com

**Keywords:** vehicular networks, artificial intelligence, security, Internet of Vehicles, datasets

## Abstract

Vehicular networks have become a critical component of modern transportation systems by facilitating communication between vehicles and infrastructure. Nonetheless, the security of such networks remains a significant concern, given the potential risks associated with cyberattacks. For this purpose, artificial intelligence approaches have been explored to enhance the security of vehicular networks. Using artificial intelligence algorithms to analyze large datasets can enable the early identification and mitigation of potential threats. However, developing and testing effective artificial-intelligence-based solutions for vehicular networks necessitates access to diverse datasets that accurately capture the various security challenges and attack scenarios in this context. In light of this, the present survey comprehensively examines the vehicular network environment, the associated security issues, and existing datasets. Specifically, we begin with a general overview of the vehicular network environment and its security challenges. Following this, we introduce an innovative taxonomy designed to classify datasets pertinent to vehicular network security and analyze key features of these datasets. The survey concludes with a tailored guide aimed at researchers in the vehicular network domain. This guide offers strategic advice on selecting the most appropriate datasets for specific research scenarios in the field.

## 1. Introduction

**Figure 1:**
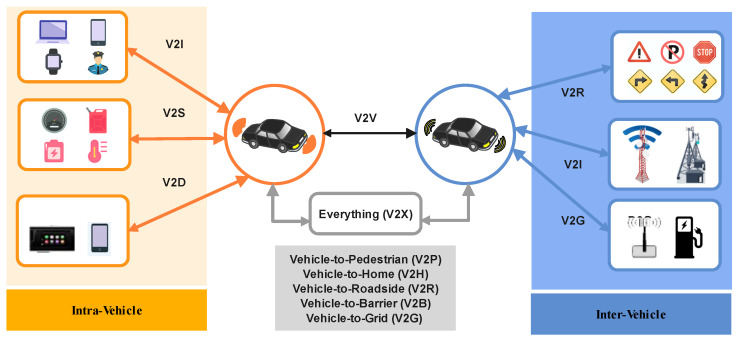
Vehicular communication scheme: intra-vehicle and inter-vehicle Iinteractions.

A vehicular network is a variant of a communication network that connects and brings together vehicles and roadside infrastructures within Intelligent Transportation Systems (ITSs). Vehicles are equipped with smart devices connected to the network that detect vehicles, update and store the driving status, and identify communications with other vehicles and the internet. Indeed, each vehicle in the network is equipped with communication-supporting devices such as Event Data Recorders (EDRs) and sensors. Sensors collect vehicle information (e.g., location, speed, and acceleration) and share it with the neighboring vehicles and adjacent roadside units (RSUs) using wireless interfaces (e.g., Long-Term Evolution (LTE) and Dedicated Short-Range Communication (DSRC)). DSRC operates on the 5.9 GHz frequency band, providing high-bandwidth, low-latency communication between vehicles and infrastructure. These types of equipment communicate over multiple in-vehicle networks. For instance, the vehicle comprises 100 to 200 electronic control units (ECUs) communicating across multiple network segments using Ethernet, FlexRay, CAN, and wireless technologies, such as LTE, Bluetooth, Wi-Fi, and other proprietary technologies. The use of such developed communications technologies in vehicles is anticipated to ensure seamless connectivity to various existing networks. However, in contrast with vehicular ad hoc networks (VANETs), which have a limited number of types of communication, the Internet of Vehicles (IoV) allows several smart devices to connect to the network and makes the network huge and scalable. It allows vehicles to be able to connect to everything V2X (Vehicle-to-Everything) [1] and share knowledge about the vehicle and its surroundings. Generally, V2X communication can be divided into in-vehicle or intra-vehicle networks and inter-vehicle networks (see Figure 1). The intra-vehicle communication models encompass Vehicle-to-Sensors (V2S) [2], Vehicle-to-Driver (V2D) [3], and Device-to-Device (D2D). Meanwhile, inter-vehicle interaction models are established to connect the vehicle with the environment. In particular, this communication model includes V2V, V2I, Vehicle-to-Pedestrian (V2P), Vehicle-to-Home (V2H), Vehicle-to-Roadside (V2R), Vehicle-to-Barrier (V2B), and Vehicle-to-Grid (V2G) [4] models. Owing to the continuous progression of vehicular networks [5], security becomes of paramount importance because of the direct effects on user safety.

Although vehicular networks have an open nature to the environment for connectivity, this has led to several security issues and challenges in communication reliability. Therefore, V2X systems became more prone and vulnerable to cyber-attacks where they could easily be hacked. There are diverse vehicular network security issues and threats that come from various types of inter-element communication, embedded sensors, system architecture, mobility, and the network’s real-time operational characteristics. Therefore, security in the vehicle is highly dependent on the medium used for communication, operation, and infrastructure. Ultimately, the delays in information transmission may lead to multiple privacy issues [6]. Thus, attackers may influence the vehicles, the user safety, and the driving track if they successfully take access.

A vehicular network is a variant of a communication network that connects and brings together vehicles and roadside infrastructures within Intelligent Transportation Systems (ITSs). Vehicles are equipped with smart devices connected to the network that detect vehicles, update and store the driving status, and identify communications with other vehicles and the internet. Indeed, each vehicle in the network is equipped with communication-supporting devices such as Event Data Recorders (EDRs) and sensors. Sensors collect vehicle information (e.g., location, speed, and acceleration) and share it with the neighboring vehicles and adjacent roadside units (RSU) using wireless interfaces (e.g., Long-Term Evolution (LTE) and Dedicated Short-Range Communication (DSRC)). DSRC operates on the 5.9 GHz frequency band, providing high-bandwidth, low-latency communication between vehicles and infrastructure. These types of equipment communicate over multiple in-vehicle networks. For instance, the vehicle comprises 100 to 200 electronic control units (ECUs) communicating across multiple network segments using Ethernet, FlexRay, CAN, and wireless technologies, such as LTE, Bluetooth, Wi-Fi, and other proprietary technologies. The use of such developed communications technologies in vehicles is anticipated to ensure seamless connectivity to various existing networks. However, in contrast with vehicular ad hoc networks (VANETs), which have a limited number of types of communication, the Internet of Vehicles (IoV) allows several smart devices to connect to the network and makes the network huge and scalable. It allows vehicles to be able to connect to everything V2X (Vehicle-to-Everything) [1] and share knowledge about the vehicle and its surroundings. Generally, V2X communication can be divided into the in-vehicle or intra-vehicle network and the inter-vehicle network. The intra-vehicle communication models encompass Vehicle-to-Sensors (V2S) [2], Vehicle-to-Driver (V2D) [3], and Device-to-Device (D2D). Meanwhile, inter-vehicle interaction models are established to connect the vehicle with the environment to address these issues; automated monitoring systems and frameworks based on artificial intelligence (AI) have been a promising solution in detecting attacks on the IoV [7]. Specifically, researchers have shown great interest in V2X network attack detection [8] using solutions based on machine learning (ML) [9] and deep learning (DL) [10]. These different AI approaches and frameworks are tested and validated based on a trained model. The authors of [11] discussed the importance of leveraging ML in IoV security risk mitigation and trust management. It provides an in-depth examination of the usage of ML for these potential threats using ML techniques. These trained models need a huge source of vehicular information. For example, the authors of [12] proposed a Zero-X, a cutting-edge security framework designed for the IoV, addressing the mounting cybersecurity threats amplified by 0-day attacks. The evaluations on recent datasets validate the efficacy in detecting various attack types while maintaining a minimal false positive rate. Hence, ML emerges as a promising solution for attack detection, offering the capability for a single framework to detect multiple attack types while validating its effectiveness across diverse attack scenarios. Meanwhile, ML has been integrated with various other technologies to achieve similar objectives. For instance, in [13], the authors conducted a thorough examination of cyber threats targeting Connected Autonomous Vehicles (CAVs), encompassing issues such as inter- and intra-vehicular communication, ML, and quantum-computing-based attacks. This study not only assesses the effectiveness of existing countermeasures but also proposes additional strategies to empower CAV cybersecurity. Moreover, it delves into the implications of emerging technologies like ML, federated learning, and blockchain on CAV security, offering insights into risk mitigation measures. With this wide adoption of these different techniques, there are limited resources and information, and most of them are private. Therefore, available datasets and open resources represent a big challenge for IoV security solutions. This challenge highlights the need for more research studies that focus on assessing and surveying public datasets for vehicular network security. The limited number of studies that concentrate on vehicular network datasets, especially in the context of vehicular network security, may significantly undermine the efficacy of security solutions. Furthermore, prior investigations have been proposed with a need for more precision to particular resources and targets. They should have conducted an exhaustive analysis of the available intra- and inter-vehicular environment security datasets. Therefore, the primary objective of this review paper is to undertake a critical study and review of the existing datasets in IoV-based solutions for security enforcement. Furthermore, this study provides a comprehensive exposition of the different datasets used for IoV network security enforcement solutions. In particular, this communication model includes V2V, V2I, Vehicle-to-Pedestrian (V2P), Vehicle-to-Home (V2H), Vehicle-to-Roadside (V2R), Vehicle-to-Barrier (V2B), and Vehicle-to-Grid (V2G) [4] models. Owing to the continuous progression of vehicular networks [5], security becomes of paramount importance because of the direct effects on user safety.

Despite the fact that vehicular networks maintain open connectivity with their environment, this openness has introduced numerous security issues and challenges in ensuring reliable communication. Therefore, V2X systems have become more prone and vulnerable to cyber-attacks, where they could easily be hacked. There are diverse vehicular network security issues and threats that come from various types of inter-element communication, embedded sensors, system architecture, mobility, and the network’s real-time operational characteristics. Therefore, security in the vehicle is highly dependent on the medium used for communication, operation, and infrastructure. Ultimately, the delays in information transmission may lead to multiple privacy issues [6]. Thus, attackers may influence the vehicles, the user safety, and the driving track if they successfully make access.

This paper intends to highlight vehicular network security datasets and help researchers select the best datasets for their work. The study’s specific goals are as follows:

- A brief vehicular network background is presented.
- The existing inter- and intra-vehicular network communication datasets are introduced.
- A detailed taxonomy of possible intra-vehicular and inter-vehicular communication dataset categories is proposed.
- A guide to selecting suitable vehicular network security datasets for research needs is provided.

The remainder of this paper is organized as follows: Section 2 provides related works on vehicular networks datasets. Section 3 outlines a brief overview of the vehicular network environment. Next, Section 4 describes the primary taxonomy of the two types of vehicular network datasets for security, and we discuss the possible classifications. Section 5 presents the existing inter-vehicular communication datasets. Then, Section 6 addresses the intra-vehicular communication datasets. Finally, there is a discussion in Section 8 that guides future researchers to select the most suitable dataset, and Section 9 concludes the paper. Table 1 lists the notations used in the paper.

## 2. Existing Surveys on Vehicular Networks Datasets

The use of datasets in vehicular network research has several benefits. First, datasets provide a way to evaluate the performance of vehicular network protocols and algorithms in a realistic and repeatable manner. Second, datasets can be used to develop and validate models of vehicular traffic, which can be used to predict traffic patterns [14] and plan infrastructure improvements. Finally, datasets can be used to develop new applications and services that can improve safety, reduce congestion, and enhance the driving experience. In this paper, we will review the current state of the art used in the context of vehicular network datasets.

Despite the growing demand for datasets in vehicular network research, only a few surveys comprehensively review and compare the existing datasets. In their review of datasets in the context of V2X security, the authors in [15] analyzed and classified the datasets based on their targeted architecture, the types of attacks included in each dataset, and their severity. While their approach is commendable, their review of the existing datasets could be more exhaustive. Additionally, some metrics, such as the severity of attacks in the datasets, need to be clearly defined or explained. The authors of [16] proposed a review to analyze intrusion-detection datasets for automotive systems and highlighted the requirements for such datasets. However, they did not define the existing datasets. Additionally, despite the review’s focus on vehicular security, the authors did not consider the attacks integrated into datasets and their effects. Another study in [17] comprehensively assessed the datasets in the context of automotive IDS. The authors considered various aspects, including the nature of the datasets, their environment, and the complexity of the covered attacks, when comparing them. The paper employed a quantitative metric to assess the dataset balance and the coverage of attacks, complemented by a qualitative evaluation of existing datasets in the field of automotive IDS. In the same context, the authors of [18] focused on IDS datasets within organizational security frameworks. The paper addresses the rising frequency and severity of network attacks by emphasizing the importance of continual monitoring and analysis. Intrusions, defined as attempts to compromise computer networks’ confidentiality, integrity, or availability, are the primary concern. This paper provides an overview of recent IDS advancements, discusses future research directions for detecting malicious operations, and includes detailed descriptions of publicly available datasets and intrusion-handling strategies.

These few studies are classified based on the studies datasets in Table 2.

## 3. Vehicular Networks: Background

This section presents brief background knowledge to readers about vehicular network’s generic key concepts and security threats.

### 3.1. Vehicular Networks Environments

As a major contributor to smart cities, vehicular networks succeed in making it simple to improve passenger safety and satisfaction using short-range wireless communication. These networks primarily encompass vehicular ad hoc networks (VANETs), the Internet of Vehicles (IoV), and Vehicular Computing Networks (VCNs).

A VANET is a self-organized network that links vehicles to improve driving safety and traffic management with internet access [39]. It supports V2V and V2I communications with the RSU installed along the roadside at intersections or parking spaces. Vehicles will have an onboard unit (OBU) to exchange data. In addition, they will have a resource command processor (RCP), read/write memory for storing and receiving data, a user interface, a specialized surface for connecting to other OBUs, and a network device for wireless communication. All VANET activities aim to spread road safety information across nodes. Thus, frequent data exchange on the network necessitates security.

The IoV, as a special case of the Internet of Things (IoT) [40], represents a new paradigm driven by recent advancements in vehicular networking and communications. Foremost, IoVs are quickly moving towards a context-awareness system, as are their environments. Thus, they become increasingly capable of detecting, processing, and communicating thanks to the quick adoption of cellular vehicle-to-everything (C-V2X) [41]. In addition, vehicles may communicate with the environment and IoT devices to collect a vast amount of road data for each vehicle. Indeed, IoV systems are equipped with sensors that yield information uploaded as filtered sensed data to centralized processing equipment or computation units for computation and analysis. This analysis aims to provide performance metric functions and optimize the Quality of Service (QoS) through exchanging sensor inputs among vehicles. Therefore, vehicular systems rely on direct line of sight for context awareness owing to the exchange of driving environmental information via Basic Safety Messages (BSMs). The BSMs can carry details about the vehicle’s current position, speed, and direction, among other things, and provide valuable support for vehicular communication.

Finally, VCNs [42] refers to the integration of computing, communication, and vehicular technologies to provide advanced services and applications for drivers and passengers in vehicles. Vehicles in VCNs can communicate with each other directly or through intermediate vehicles or new paradigms or technologies. Indeed, fog, cloud, and edge computing are three major paradigms that play a crucial role in VCNs. They allow the sharing of information about traffic conditions, road hazards, and other relevant data to provide real-time traffic updates. Fog computing offers low-latency and real-time services; cloud computing delivers scalable and cost-effective storage and processing; edge computing provides low-latency and real-time processing capabilities. Combining these paradigms can enable VCNs to provide advanced services and applications for vehicle drivers and passengers, such as autonomous driving, intelligent traffic management, and personalized in-vehicle services.

### 3.2. Vehicular Network Architecture

The vehicular network’s architecture is organized into three distinct layers, establishing a comprehensive framework for its secure operation and security. The sensing layer, the base of this architecture, is composed of vehicle sensors, such as radar, camera, and lidar. Its main responsibility is gathering vital real-time data essential to vehicle functioning. Nonetheless, there are many security issues with this layer. It is especially vulnerable to sophisticated eavesdropping and spoofing attacks, which seriously compromise the confidentiality and authenticity of the sensor data and could impact decision-making. Above the sensing layer is the communication layer, which serves as the base for all vehicle communication. It is an efficient link between internal (intra-vehicular) and exterior (inter-vehicular) communication channels, essential for the effective and harmonic data flow between vehicles and other roadside infrastructure components. Even with its importance, this layer is not risk-free. It is vulnerable to various cyber threats, such as malicious data tampering and eavesdropping. It also inherits threats from the sensing layer and may even amplify them, emphasizing the necessity of powerful, multi-layered security measures. At the top of the hierarchical structure lies the control layer, which manages highly developed automated vehicle control systems, including critical functions such as steering and speed control. The robustness and reliability of the underlying sensing and communication layers are directly linked to the efficiency and safety of this layer. Any degradation of these fundamental layers might seriously affect overall safety and vehicle control.

### 3.3. Communication Layer

#### 3.3.1. Intra-Vehicular Communication Protocols

This section will enumerate different protocols utilized within in-vehicle networks (see Table 3), highlighting their adaptability to vehicular networks. These protocols address various aspects of automotive functionality, ranging from control systems to infotainment, and illustrate the diverse technological landscape required to support modern vehicle architectures.

A Controller Area Network (CAN) is an asynchronous serial bus network that links devices, sensors, and actuators within a system or sub-system for control purposes [43]. A CAN is a multi-master communication protocol primarily designed for data integrity and automotive applications, supporting data rates of up to 1 Mb/s. Beyond its automotive use, a CAN is also a versatile embedded communication system for microcontrollers and industrial control systems. In recent years, there have been two primary physical-layer designs for CAN: high-speed and low-speed CAN. Both utilize differential voltage on wire pairs for communication, offering data transfer rates of up to 1 Mb/s for high-speed CAN and up to 125 kb/s for low-speed/fault-tolerant CAN. CAN is recognized for its affordability and high reliability, making it a popular choice in powertrain, chassis, and body electronics. However, its limited bandwidth and shared medium for data transmissions restrict its application in domains like infotainment.

A Local Interconnect Network (LIN) complements CANs by using universal asynchronous receiver–transmitter technology in a single-master, multiple–slave configuration. LINs offer a cost-effective solution for connecting a vehicle’s motors, switches, and sensors. The controller node in LINs connects individual sensors and actuators to higher-level networks like CANs. Compared to CANs, LINs have several distinctive features: they provide efficient communication for sensors and actuators that do not require the bandwidth and flexibility of CAN, act as a cost-effective sub-network alongside CAN, can be implemented with standard asynchronous communication interfaces, and do not involve any protocol license fees as they are an open-source protocol. Consequently, LINs are commonly used in body electronics due to their cost-effectiveness and straightforward bandwidth requirements.

In contrast, FlexRay is designed for high-demand systems, offering a dual-channel data rate of up to 10 Mb/s and catering to critical safety systems like brake-by-wire. FlexRay can also support in-vehicle networks, working alongside protocols like CAN and LIN. Regarding cost reduction, FlexRay can replace multiple CAN networks to meet vehicle bandwidth requirements. It is widely regarded as the next-generation in-vehicle networking technology, facilitating the management of new safety and comfort features. Unlike CAN, FlexRay delegates error correction to its application layer without an error recovery mechanism. Despite its higher cost than CAN, FlexRay is used in high-performance applications within powertrain and safety systems, including active suspension and adaptive cruise control.

Further expanding on the need for a high data rate, Media-Oriented System Transport (MOST) technology utilizes a ring topology to synchronize audio and video signals, supporting up to 64 devices. MOST stands out with its impressive data rate, reaching up to 50 Mb/s and supporting up to 64 MOST devices in a ring configuration [44]. Alternative topologies like double rings are also possible, especially for safety-related applications. While MOST offers a higher bandwidth than CANs, LINs, and FlexRay, it comes at a significantly higher cost. As a result, MOST is typically recommended for in-vehicle camera and video connections.

In addition, Low-Voltage Differential Signaling (LVDS) is another technology that can be integrated into modern automotive electronics [45], characterized by its high data transmission speeds and robust resistance to noise. This signaling method, which operates at low voltages, is ideal for automotive applications that prioritize efficiency. It effectively reduces electromagnetic interference, allowing vital vehicle systems like navigation, infotainment, and ADAS to run without interruption. LVDS also reliably delivers high-resolution video data reliably across extended cable lengths, making it essential for applications like rear-view cameras and vehicle display panels. By incorporating LVDS, vehicle manufacturers can significantly enhance the performance and safety of their models, addressing the rising consumer demand for advanced connectivity and autonomous driving features.

Lastly, Automotive Ethernet has long been the dominant technology for local area networks, playing a pivotal role in shaping various forms of communication [46]. When applied to the automotive domain, it is known as Automotive Ethernet and is the foundation for connecting components within a vehicle. Originally designed to meet a range of requirements, including electricity, bandwidth, latency, synchronization, and network management considerations, Automotive Ethernet presents several significant advantages. One of the most prominent benefits is the substantial increase in communication bandwidth, which greatly enhances the capabilities of advanced driving functions and infotainment systems. Furthermore, it redefines the structure of in-vehicle networks, shifting from previously decentralized, domain-specific topologies to more efficient hierarchical ones.

#### 3.3.2. Inter-Vehicular Communication

Inter-vehicular communication protocols enable vehicles to communicate with one another and the surrounding infrastructure.

In this section, we explore the dynamic landscape of these protocols, starting with Dedicated Short-Range Communication (DSRC), which uses specific frequency bands for vehicle communications as outlined by the Federal Communications Commission (FCC) [47,48]. DSRC is organized into seven 10 MHz channels, currently spanning the frequency spectrum from 5.850 to 5.925 GHz, within bands numbered between 172 and 184. Notably, channel 178 is exclusively designated for secure communications and is referred to as the control channel, while the remaining four service channels, numbered 174, 176, 180, and 182, are intended for reporting insecure situations. However, the first and seventh channels (172 and 184) are reserved for specific purposes.

Additionally, IEEE 802.11p was introduced as an extension of the IEEE 802.11 family of protocols to cater to the specific needs of vehicular networks, where seamless communication among vehicles and infrastructure is paramount. Within the scope of IoV, IEEE 802.11p plays a crucial role by defining the parameters and characteristics of both the physical and medium-access layers. These definitions are instrumental in ensuring reliable and efficient communication in IoV scenarios. By providing a dedicated framework for IoV, IEEE 802.11p aligns with the evolving demands of connected and Intelligent Transportation Systems.

The European Telecommunications Standards Institute Technical Committee for Intelligent Transport Systems (ETSI TC ITS) has also played an important role. The ETSI TC ITS has established a standardized architecture for the IoV with invaluable input from automotive manufacturers and organizations like the Car-to-Car Communication Consortium [49]. In this context, they have adopted the GeoNetworking (GN) protocol as a fundamental component for packet routing within the IoV framework, primarily focusing on safety applications. The primary objective of creating ETSI ITS is to propose performance enhancements for the GN protocol, which is critical for optimizing Vehicle-to-Internet communication, particularly in the context of IoV standards. The analysis hinges on simulation-based methodologies to pinpoint and rectify performance challenges, especially in IoV scenarios. The overarching aim is to ensure seamless and efficient communication between vehicles and the internet within the broader IoV standards framework.

Meanwhile, Cellular Vehicle-to-Everything (C-V2X) is a cellular-based communication technology for vehicles operating on 10 or 20 MHz channels. It utilizes LTE-like numerology, with 1 ms subframes and 180 kHz resource blocks. Data are transmitted in Transport Blocks (TBs) over Physical Sidelink Shared Channels (PSSCH). At the same time, control information is sent in Sidelink Control Information (SCI) over Physical Sidelink Control Channels (PSCCH) in the same subframe. C-V2X provides flexibility in configuration, adapting to conditions and congestion levels. It employs turbo coding for data and convolutional encoding for SCI. Sensing-Based Semi-Persistent Scheduling (SB-SPS) helps reserve sub-channels, preventing packet collisions. HARQ retransmission enhances reliability. C-V2X enables vehicles to communicate with each other and improves infrastructure, safety, and traffic management.

SO/SAE 21434, a cybersecurity standard [50], provides essential guidance for original equipment manufacturers (OEMs) and suppliers in the automotive industry to manage cybersecurity risks within electrical and electronic (E/E) systems of road vehicles. While ISO/SAE 21434 does not explicitly focus on the IoV, its principles and framework are highly relevant in the context of IoV networks. ISO/SAE 21434’s emphasis on risk assessment, threat analysis, security controls, and incident response aligns with the need for robust cybersecurity in IoV networks. It provides a foundation for managing and mitigating cybersecurity risks within vehicles, which are integral components of the IoV. The standard encourages a proactive approach to safeguarding the integrity, confidentiality, and availability of data and services within IoV networks.

Finally, ISO 21210, ref. [51] part of the Continuous Air interface, Long- and Medium-range (CALM) series, within ISO Technical Committee 204 Working Group 16 (ISO TC204 WG16), defines how IPv6 network protocols and services enhance the global connectivity of Intelligent Transport Systems stations. This standard details how IPv6 facilitates the uninterrupted network connectivity required for ITS stations to function effectively as access routers, allowing both fixed and mobile units, such as vehicles and roadside infrastructure, to connect to the internet. It does not introduce new protocols or data structures. Still, it focuses on how existing IPv6 protocols from the Internet Engineering Task Force (IETF) can be utilized to maintain robust connectivity among ITS stations. Additionally, ISO 21210 outlines how legacy systems can integrate within this framework, ensuring backward compatibility and facilitating integration into advanced networked environments.

### 3.4. Vehicular Network Sensors Used for Data Collection

Vehicular network sensors [52] play a crucial role in generating datasets for vehicular security. This section presents the different vehicular sensors used for vehicular security datasets. We divide these sensors into two categories: in-vehicle sensors and road sensors.

- In-vehicle sensors: In-vehicle sensors are located within the car and measure various aspects of a vehicle’s performance, such as acceleration, braking, and impacts, using accelerometers [53]. Gyroscopes measure a vehicle’s orientation and movement, while ABS sensors detect wheel lockup during braking. Additionally, engine speed sensors measure the engine’s rotational speed, while lane departure warning sensors monitor the vehicle’s position within a lane. Blind spot monitor sensors, another type of in-vehicle sensor, detect the blind spots of other vehicles in the car.
- Road Sensors: Road sensors are located on the road and measure traffic and environmental conditions to improve vehicular security [54]. Road sensors include traffic cameras that capture images of traffic flow, traffic light sensors that detect the presence of vehicles at intersections, and weather sensors that measure environmental conditions such as temperature and precipitation. In addition, road surface sensors detect road surface conditions such as temperature and friction, while pedestrian sensors detect the presence of pedestrians at crosswalks. These sensors work together to generate datasets that can improve the safety of drivers, passengers, and pedestrians by detecting hazardous conditions and warning drivers to take caution. In summary, in-vehicle and road sensors are crucial components of vehicular security. Their data are essential in creating datasets that can improve the safety of vehicles on the road.

These sensors work together to generate datasets that can be used to improve vehicular security. As described in [55], in-vehicle sensors can detect when a driver behaves erratically and takes corrective action. In contrast, road sensors can detect hazardous conditions and warn drivers to take caution. In short, in-vehicle and road sensors are essential components of vehicular security. Their data are critical in creating datasets that can improve the safety of drivers, passengers, and pedestrians.

### 3.5. Vehicular Network Security Challenges

**Figure 2:**
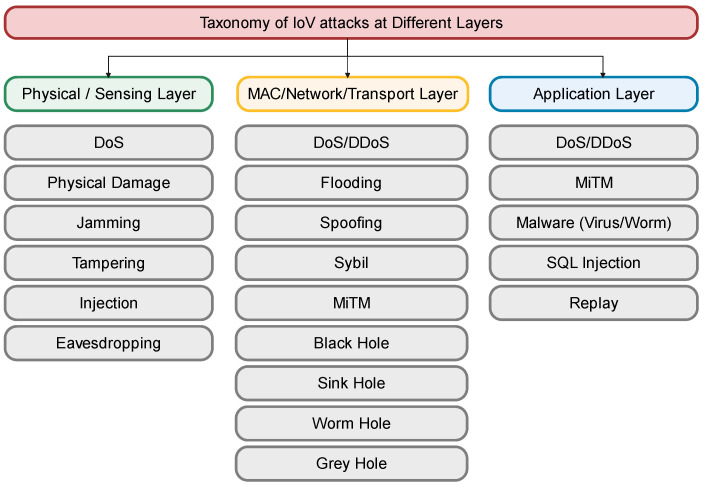
Performed attacks on IoV at different layers.

Since the global scheme is designed for a V2X network, there is usually no reliable infrastructure. Vehicles communicate wirelessly using an open channel on the internet. The participating entity’s openness and communication technologies bring challenges such as security vulnerabilities, data privacy, transparency, scalability, and data integrity. Moreover, the absence of centralized supervision, coupled with the crucial features of network security required to ensure secure transmission during information sharing, has led to various challenges. As a result, attackers may influence the vehicle and the user. However, it is necessary to address the existing attacks to enhance IoV security. Different classes of vehicular network security threats incorporate various attacks that can occur. These attacks can be classified based on their target layer (Figure 2 depicts this classification).

In vehicular communication systems, security threats pose significant risks across different layers of the communication stack. Vehicles are vulnerable to various attacks at the physical or sensing layer to disrupt their basic functionalities. These attacks directly affect the vehicle software or hardware via access control [56] to cause physical vehicle damage attack [57]. DoS attacks can overwhelm vehicle sensors while jamming interferes with the wireless communication channels, rendering them unusable. Injection attacks may introduce false data, and tampering physically alters device operations or data integrity. Moreover, eavesdropping threats enable the unauthorized interception of sensitive information exchanged by the vehicles. Moving up to the network or transport layer, the threats evolve into more sophisticated forms. DoS and DDoS attacks [58] can flood the network with traffic, disrupting communication across vehicles and infrastructure. The following attacks can track a vehicle’s broadcast messages. The Sybil attack [59] undermines the system by creating many fictitious nodes, while spoofing [60] manipulates communication by impersonating another device. MiTM attacks [56] secretly relay and possibly alter the communication between two parties who believe they are directly communicating with each other. Additionally, various hole attacks [57,61] such as blackhole, sinkhole, wormhole, and greyhole, exploit network protocols to disrupt the routing of packets, leading to information loss or delay. GPS deception attacks [62], masquerading attacks [56], wormhole attacks [61], cookie theft attacks [63], channel interference attacks [62], and route modification attacks [56] gave become even more cunning and deceitful at the application layer, which interfaces directly with the user. Injection attacks [61] compromise the system by inserting malicious data into a stream of legitimate data. Malware [61], i.e., malicious software, can disrupt or damage the vehicle’s system operation. Replay attacks [64] involve the interception and retransmission of valid data streams, potentially to gain unauthorized access or services. Each of these threats can have dire consequences on the reliability and safety of vehicular communications, necessitating robust security measures at every layer to safeguard against these potential attacks. These attacks are assessed and evaluated in Table 4.

## 4. Intra-/Intra-Vehicular Communication Datasets for Vehicular Network Security: Taxonomy

**Figure 3:**
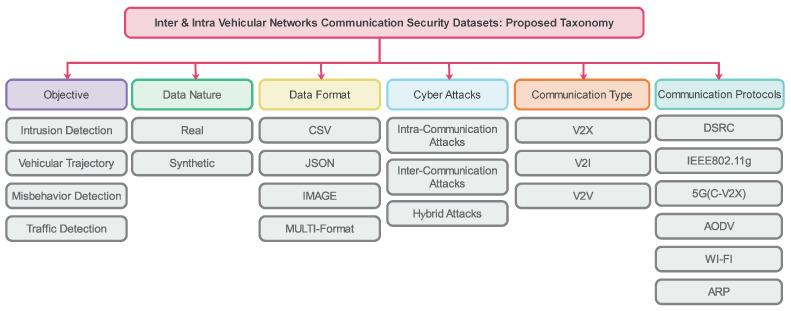
Intra- and inter-vehicular communication datasets for vehicular networks security: proposed taxonomy scheme.

This section proposes a detailed taxonomy for intra- and inter-vehicular communication datasets for vehicular network security. It is broadly classified into six categories, as shown in Figure 3. The complete taxonomy is described as follows:

- Objective of the dataset: We list the dataset’s goal and usability aspects. There are four significant usage objectives in vehicular network communication datasets: datasets for intrusion detection, datasets for misbehavior detection, datasets for vehicle trajectory prediction, and datasets for traffic scenario analysis.
- Data Nature: We highlight the general nature of the data used, which mainly belong to two types: real data from real-life scenarios and a simulated dataset, named “synthetic”, from the simulation frameworks.
- Data Format: We describe all of the existing data structures within the database. There are different types, including comma-separated values (CSV)s, JavaScript Object Notation (JSON), and image format. In addition, various datasets may encompass different types of data or multi-formats in one global file.
- Cyber Threats: We discuss various types of attacks in three major sectors, including all possible attacks in inter-vehicular, intra-vehicular, and hybrid communications.
- Communication Types: We describe three types of communications: V2X, V2V, and V2I. These three communication types are the most prone to connectivity in vehicular network communication links. They represent an easy medium between the vehicle and the attacker, so there are different datasets for vehicular network communication security for all three communications types.
- Communication Protocols: We list the different serial port communication protocols in the existing datasets, including DSRC, IEEE 802.11 g, 5G(C-V2X), AODV, WI-FI, and ARP.

## 5. Inter-Vehicular Communication Datasets

To better understand and mitigate the IoV security threats, researchers have developed numerous datasets specifically focused on inter-vehicular communication in the context of security. These datasets can help to build more secure vehicular communication protocols and applications. This section details the different inter-vehicular datasets used in the context of IoV security (See Table 5).

### 5.1. VeReMi Dataset

VeReMi [19] is a widely used dataset for assessing IoV misbehavior, designed to emulate dynamic smart vehicle scenarios and integrate key safety features such as position, distance, speed, and arrival angles. It is generated using LuST, VEINS, and Maat, and comprises message logs from onboard vehicles in a simulated Luxembourg City Vehicle Network (LCvehicular network). This network is part of a smart city ecosystem featuring IoT, AI, autonomous vehicles, and more, offering various vehicle densities and attack scenarios. The VeReMi workflow begins by setting up and running traffic simulations to see how vehicles communicate during both normal and attack scenarios. This produces message logs, which are then checked for any unusual activities using special algorithms. After identifying any issues, the data are analyzed and turned into graphs. The final step involves sharing the results and data through publications. VeReMi includes 225 simulations evaluating IoV security under different conditions, featuring diverse attacker, traffic, and vehicle densities. The dataset explores several attack types, including random, random offset, eventual stop, constant, and constant offset, each posing unique security challenges. This structured workflow helps ensure the safety and reliability of vehicle communication systems within complex urban environments.

### 5.2. VeReMi Extension Dataset

The author of [20] extended the VeReMi dataset to create the VeReMi Extension, enhancing it with a sensor error model, more attack scenarios, and a larger dataset. VeReMi Extension’s generation follows a similar process to VeReMi, utilizing the Framework for Misbehavior Detection (F2MD) for effective misbehavior identification. To reflect real-world scenarios, the dataset incorporates sensor error models in key data fields: Position, Velocity, Acceleration, and Heading. It introduces new attacks, distinguishes between malfunctions and attacks, and comprehensively evaluates IoV security. Attacks include DoS, data replay, disruptive, eventual stop, and traffic congestion Sybil attacks, covering many scenarios.

### 5.3. NGSIM Datasets

The data in the NGSIM datasets [21] were first gathered by cameras and then taken out of the videos that were recorded. Each sample in the track is taken every 0.1 s and comprises different pieces of data, like the type of vehicle, the current speed and acceleration, the longitudinal and lateral positions, and the length of the vehicle. There are a total of four datasets, and one of these collections is the US-101 trajectory dataset. This dataset contains six lanes, and it was collected on a 640 m long segment in Los Angeles, California, around Lankershim Avenue on the southbound US-101 highway. These data were specifically collected on 15 June 2005, between 7:50 a.m. and 8:35 a.m. In addition, the NGSIM dataset includes an I-80 trajectory dataset that traces the trajectory data of six lanes, including a high-occupancy vehicle (HOV) lane. It was obtained from a 500 m long segment of the I-80 freeway in Emeryville (San Francisco), CA, USA. The data were collected on 13 April 2005, during two time periods, including 15 min from 4:00 p.m. to 4:15 p.m. and 30 min from 5:00 p.m. to 5:30 p.m. The third dataset is a Peachtree trajectory dataset composed of five intersections (four are signalized and one is not) and two or three through lanes in each direction. It was collected on a 640 m long segment of Peachtree Street in Atlanta, Georgia, on 8 November 2006. This dataset consists of two 15 min periods: 12:45 p.m. to 1:00 p.m. and 4:00 p.m. to 4:15 p.m. The last dataset is the Lankershim trajectory dataset, which contains three or four lanes and four signalized intersections. This dataset was collected on a 488 m long segment of Lankershim Boulevard in the Universal City neighborhood of Los Angeles, CA, USA. The data were collected for 30 min from 8:30 a.m. to 9:00 a.m. on 16 June 2005

### 5.4. PeMS Dataset

The Caltrans Performance Measurement System (PeMS) is the primary data source, collecting real-time data from approximately 40,000 detectors installed throughout California’s major metropolitan regions. In addition to providing real-time data, PeMS serves as an Archived Data User Service (ADUS), offering over ten years of historical data for in-depth analysis. PeMS is a comprehensive platform that integrates diverse information from Caltrans and local agency systems. This includes data from traffic detectors, incident reports, lane closures, toll tag information, census traffic counts, vehicle classification, weight in motion, and roadway inventory. This wealth of information aids researchers and policymakers in making informed decisions related to transportation planning, infrastructure development, and traffic management, ultimately enhancing the overall transportation experience.

### 5.5. HighD Dataset

The highD dataset is a recently compiled collection of real vehicle movements documented on highways in Germany. A drone addressed common obstacles encountered in traditional traffic data collection methods, such as obstructions, by providing an aerial perspective. The dataset comprises recordings of more than 110,500 cars in six locations, with an average recording duration of 17 min (totaling 16.5 h) covering a road segment of approximately 420 m. Each vehicle’s trajectory, size, type, and maneuvers were automatically extracted, and the median visibility duration for each car was 13.6 s. The primary addition in the HighD dataset is the extraction of predefined maneuvers detected based on a set of rules and thresholds for each vehicle. The list of identified driving maneuvers in the HighD dataset consists of four distinct types. The first is “Free Driving”, which refers to driving without being influenced by a vehicle ahead. The second is “Vehicle Following”, which involves actively tracking another vehicle on the road. The third is “Critical Maneuver”, which occurs when the Time to Collision (TTC) or Time Headway (THW) with a preceding vehicle is low. The final category is “Lane Change”, which involves crossing lane markings and moving to a different lane on the road.

### 5.6. Warrigal Dataset

This extensive database was gathered in an industrial and manufacturing setting from trucks and smaller 4WD vehicles. It is collected from 13 vehicles operating in a large quarry-like setting over a time frame of three years. The dataset comprises information about the vehicles’ state, including their position, speed, and direction, as well as information about their peer-to-peer radio communications. The data were recorded using a Fastrax IT321 GPS chip for position tracking over three years, with a 1-hertz resolution. The dataset primarily consists of two major components: vehicle positions and radio communications. Regarding communication, the authors of the dataset categorize vehicles into two types based on the frequency used: Light vehicles are equipped with a single 2.4 GHz antenna and a single 433 MHz antenna, while heavy vehicles are equipped with a single 2.4 GHz antenna and a pair of 433 MHz antennas. The dataset is organized into daily periods and labeled with the year, month, and day. Additionally, the files are categorized by the type of information they contain, such as state, communication, signal strength, vehicle types, and map.

### 5.7. The DeepSense 6G Dataset

This dataset comprises multiple scenarios, each of which is an independent dataset containing multi-modal sensing and communication data specific to that scenario. The data collected in each scenario reflect a real deployment situation and can serve one or more applications. The DeepSense 6G dataset encompasses a diverse range of deployment scenarios, including V2I, drone communication, V2V, Reflective Intelligent Surfaces (RIS), indoor use cases, pedestrian scenarios, and more. The diverse range of scenarios highlights the considerable capacity of the DeepSense 6G dataset to support a variety of applications and use cases. Therefore, DeepSense 6G is a valuable resource for carrying out research and promoting 6G technology innovation. To compile this dataset, various sensing devices, such as mmWave receivers, RGB cameras, GPS RTK kits, LiDAR, and radar, were employed.

### 5.8. VDoS-LRS Dataset

VDoS-LRS dataset introduced in [26] represents Vehicular Denial of Service Networks and the Systems Laboratory. It comprises two sets of data, capturing both normal and malicious traffic. VDoS-LRS includes data related to three types of Denial of Service (DoS) attacks. The first type is SYN Flood, exploiting TCP protocol vulnerabilities by inundating the vehicle with numerous SYN requests, aiming to deplete its resources and render it unusable for legitimate vehicles. The second category is UDP Flood, a type of attack overwhelming the targeted host with IP packets containing UDP datagrams, flooding random ports, and causing system overload, resulting in “Destination Unreachable” responses. The third type is the Slowloris attack, operating at the application layer. The dataset underwent evaluation in three distinct settings: urban, highway, and rural, each exhibiting unique characteristics. The researchers also considered varying vehicle speeds for each environment, with average speeds of 40 km/h in urban areas, 90 km/h on highways, and 30 km/h in rural settings.

### 5.9. Iqbal’s Dataset

Iqbal et al. [27] proposed a new dataset to identify and prevent replay attacks and false-information attacks. Various characteristics are outlined for detecting anomalies in different scenarios. For instance, for replay attacks, the message sequence number can be examined, and the expected delivery time of a message can be taken into account. It suggests a possible replay attack if the time exceeds the expected duration. Similarly, for false-information attacks, changes in position are essential. To identify fake accident reports, other nearby users can be checked for inconsistencies, and if any are found, the relevant authorities and roadside units can be alerted for further investigation. The authors used the Eclipse MOSAIC simulation framework and a set of vehicular simulation tools to model these attacks. The framework comprises different components, including OMNET++ for handling V2V and V2I communications and SUMO for simulating urban mobility.

### 5.10. VDDD Dataset

The authors of [28] proposed the VDDD dataset to simulate a VANET environment with normal and DDoS traffic. The dataset was generated using a combination of four frameworks, including SUMO, Veins, INET, and OMNeT++. To make the simulation more realistic, the King Fahad Highway, located in the Eastern Province of the Kingdom of Saudi Arabia, was used as a testbed. First, realistic network mobility traffic was generated using SUMO. Second, the SUMO mobility traffic was imported into OMNeT++ to generate the network traffic, both normal and DDoS traffic, using Veins and INET. Finally, the dataset was collected and prepared to evaluate and study the performance of several ML algorithms. For example, the VDDD scenario involves three RSUs and N vehicles along an 18 km highway with a low traffic rate of 20 nodes (N = 20) and a high traffic rate of 60 nodes (N = 60). Two levels of attack rate, 10 and 50 on the palliative performance scale (PPS), are considered. This scale is used to measure each rating scenario, resulting in four different scenarios. Additionally, one of the RSUs was configured to be the victim unit that the attack traffic would exploit.

### 5.11. Synthetic Datasets Generation for VANET Intrusion Detection

The authors of [29] have introduced a synthetic dataset tailored for intrusion detection in VANETs. This dataset allows users to configure parameters such as location, threat scenarios, the number of hosts, and the count of malicious hosts, enabling the generation of specific simulation scenarios using the NS-3 network simulator. The simulation process yields three distinct output files, including per-second routing tables, packets, and network statistics. The initial dataset provides various details about the destination, gateway, interface, and other relevant information. The second file contains information concerning TCP, UDP, IPv4, WiFi, AODV, and ARP packets. Finally, the last file includes network statistics, encompassing details such as flow IDs, TX bitrate, RX bitrate, TX packets, RX packets, lost packets, mean delay, and packet loss ratio.

## 6. Intra-Vehicular Communication Datasets

Recently, there has been a growing recognition of the necessity for security within intra-vehicular networks. This increased recognition is driven by the rising connectivity of vehicles and their growing dependence on electronic control units (ECUs). Datasets related to intra-vehicular network security hold significant value for the development of new security protocols. In this section, we will review the existing intra-vehicular networks (refer to Table 6).

### 6.1. Car-Hacking Dataset

The car-hacking dataset [30] encompasses CAN packets collected from the OBD-II port. Each CAN packet is characterized by three main features: CAN ID, representing the identifier of the CAN packet (DATA[0] to DATA[7]), indicating the 8 data bytes of the packet, and the flag, accepting two values, *T* for the injected packet and *R* for the normal packet. The dataset comprises normal traffic and three types of attacks: (1) DoS attack: A DoS packet with CAN ID“0X000” is injected every 0.3 milliseconds. (2) Fuzzy attack: Random ID and DATA values are injected every 0.5 milliseconds. (3) Spoofing Attack (RPM/gear): It injects certain CAN ID packets relevant to RPM and gear every one millisecond.

### 6.2. OTIDS Dataset

The OTIDS dataset [31] is generated by collecting CAN packets through the OBD-II. It comprises normal packets and DoS attacks with a CAN ID of “0X000”. The CSV files of fuzzy attacks do not specify whether a packet is normal or attacked. Impersonation attack CSV files are similar to fuzzy attack files. However, The Fuzzy attack injects fake CAN ID and DATA packets with random values.

### 6.3. Survival Dataset

The Human-Centered Robotics Laboratory (HCRL) has released two datasets [32] generated from three different vehicles, including the Kia Soul, Hyundai Sonata, and Chevrolet Spark. One dataset contains accurate driving records, while the other comprises anomalous driving records resulting from three attack scenarios: flooding, fuzzy, and malfunction. These attacks involved implanting attack messages every 20 s for 5 s, and each threat was captured for 25–100 s. The authors utilized the dataset to develop a detection model based on a survival analysis capable of identifying anomalies in in-vehicle networks. Survival analysis is a statistical method that focuses on the time it takes for an event to occur. This dataset was analyzed by [65], where the authors evaluated various studies utilizing this dataset. They conducted a comparative analysis, listing these studies and assessing intrusion detection using various methodologies and models.

### 6.4. SynCAN

The SynCAN Dataset [33] serves as a standard for evaluating and contrasting different CAN Intrusion Detection Systems (IDS) based on multiple attack scenarios. It comprises a training dataset and six testing datasets that comprise columns for labels, IDs, time, and signal values. The six testing datasets include test_normal.zip, which contains only normal data with a label of zero for IDS performance evaluation on unperturbed data. Other files encompass test_plateau.zip, where a signal maintains a constant value over time, and test_continuous.csv, where a signal gradually deviates from its actual value. Additionally, the dataset includes test_playback.zip, where a signal is overwritten with a recorded time series of the same signal, and test_suppress.zip, where messages of a specific ID are absent from the CAN traffic due to an attacker preventing an ECU from sending messages. For test_flooding.zip, An attacker sends messages of a specific existing ID at a high frequency to the CAN bus. The label column distinguishes between normal and intrusive data, with the training dataset’s label is always set to zero. The time column represents message timestamps, while the ID column includes IDs ranging from id1 to id10. Finally, the signal columns contain actual signal values. However, signals from different IDs may represent distinct signals, and some IDs may have a different number of signals. This dataset facilitates the unsupervised training of IDS and the assessment of their performance on both normal and abnormal data.

### 6.5. TUe v2 Dataset

The authors [34] proposed a framework for assessing IDS designed for the CAN network. They collected data from two vehicles, Opel Astra and Renault Clio, and a CAN bus prototype, which they developed to create their dataset. Additionally, they incorporated Kia Soul data from the car-hacking dataset, which is publicly available through the Eindhoven University of Technology Lab (TUe Security Group [66]). The authors initiated various attacks on the prototype to generate attack datasets, simulating these attacks on both vehicles. For a diagnostic attack, they randomly injected ten packets with CAN IDs greater than 0x700. Subsequently, they executed two fuzzing attacks involving injecting ten packets with unknown CAN IDs and altering the payload of 10 frames associated with a legitimate CAN ID. An attack replay was conducted by injecting a specific packet observed in the dataset 30 times, adjusting the timestamp to send the packets ten times faster than usual. To simulate a DoS attack, all messages within a 10 s interval were replaced by messages with a CAN ID of 0x000, sent at a rate of four packets per millisecond. Lastly, the authors simulated a suspension attack by removing all messages with a particular CAN ID over 10 s.

### 6.6. ORNL Dynamometer CAN Intrusion Dataset

The ORNL dataset [35] encompasses 12 ambient captures, providing approximately 3 h of ambient data and 33 attack captures with a total runtime of around 30 min. The data collection utilized the Kvaser Leaf Light V2, a reliable, low-cost device connected to the OBD-II port of a Linux computer. It employed SocketCAN software to gather CAN data. All data originated from a single vehicle, with the make and model intentionally undisclosed to safeguard the vehicle’s identity. The released data underwent anonymization to preserve the essential characteristics of an IDS. The vehicle was positioned on a dynamometer and actively driven during each attack. Ambient data were collected both on the dynamometer and roads, encompassing a range of normal and occasionally unconventional but benign driving actions (e.g., unbuckled seatbelt or door open while driving). The dataset categorizes recorded attacks into three types. The first category is a fuzzing attack, where frames with random IDs were injected every 0.005 s. The second category includes targeted ID fabrication and masquerade attacks. In the fabrication attack, message injection with the target ID was performed using a flam delivery technique. For the masquerading attack version, legitimate target ID frames preceding each injected frame were deleted to simulate a masquerade attack. Lastly, accelerator attacks form an additional category, exploiting a specific vulnerability related to the vehicle’s make/model, compromising the ECUs.

### 6.7. CrySyS Dataset

The CrySyS Lab created a dataset called CrySyS [36] for the SECREDAS project, which is publicly available. It contains seven short captures and one long driving scenario trace, with 20 message IDs and varying signal numbers. They also developed a signal extractor and attack generator script to complement the dataset, which can modify CAN messages in different ways, such as changing constant or random values, modifying with delta or increment/decrement values, or switching to incremented/decremented values. In addition, the attack generator can be used to simulate attacks by replacing a chosen signal with a constant value in the CrySyS traces.

### 6.8. SIMPLE Dataset

The SIMPLE dataset [37] is composed of publicly available data obtained by capturing Controller Area Network (CAN) messages from two vehicles: a 2016 Nissan Sentra and a 2011 Subaru Outback. The data acquisition utilized a Tektronix DPO 3012 oscilloscope connected through the Onboard Diagnostics II (OBD-II) port. Each driving session lasted approximately 40 min and included a mix of local and highway driving scenarios. In total, the dataset comprises over 16,000 frames and focuses on hill-climbing-style attacks.

### 6.9. Bi’s Dataset

The dataset presented in [38] is derived from various driving situations and utilizes CAN traffic obtained during the test vehicle’s daily commute route. The route covered three distinct scenarios, including country roads, highways, and congested city roads. The dataset, comprising 29,213,281 messages, encapsulates seven days of CAN traffic collected during commuter driving. It encompasses challenging road conditions such as slippery, congested, rainy, and foggy roads. To generate the attack dataset, the authors injected anomalous data into the CAN bus of the test vehicles using data injection equipment. They employed four attack models, both in the stationary and driving states, resulting in attack messages, including DoS attacks, fuzzy attacks, ulterior fuzzy attacks, and replay attacks.

## 7. Potential Features Used in Vehicular Datasets for Attack Detection

### 7.1. Inter-Vehicular Features

Features of inter-vehicle environments come from various data sources, and they help to understand and improve how traffic flows, how safe our roads are, and how efficiently we can travel. With the advancement of technologies like connected and autonomous vehicles, these features have become even more important. They help make real-time decisions, predict traffic conditions, and ensure safety. Inter-vehicular features are categorized based on their nature and application in vehicular network datasets.

- Vehicle Dynamics and Positioning: This category explores the core aspects of vehicular motion, encompassing GPS coordinates, speed, acceleration, and directional information. Such data are indispensable for deciphering vehicle behavior and analyzing movement patterns on the road. For instance, the HighD dataset is exemplary in this context. It offers a wide range of information with its detailed vehicle trajectories, speeds, and acceleration data, providing deep insights into how vehicles move and interact on highways.
- Temporal Data: Temporal data capture the chronological aspects of vehicular movements and events. They include critical timestamps of various events and the durations of specific activities or states. Data of this type are instrumental in understanding and analyzing temporal patterns and sequences over time. A notable example is the VDDD dataset, which includes event time and previous event time, allowing researchers to analyze the sequences and durations of events in a detailed manner.
- Vehicle Identification and Characteristics: This type of feature is essential for distinguishing and characterizing vehicles. It includes data on vehicle types, physical attributes, and unique identifiers. Such information is fundamental for classifying vehicles and conducting a detailed analysis of vehicle-specific behavior. The NGSIM dataset, for example, provides vehicle identification numbers and vehicle types, enabling a comprehensive study of various vehicle behaviors.
- Traffic and Congestion Analysis: In this class, the focus is on assessing traffic flow, volume, and congestion. Features include vehicle counts, traffic density measurements, and analyses of peak traffic periods. These features are vital for understanding and managing traffic congestion. The PeMS dataset, for instance, offers rich data on traffic volume, flow rate, and traffic density, which are crucial for comprehensive traffic congestion analysis.
- Network and Communication Data: This category encompasses data related to the communication networks within vehicular systems. It includes information about data packet transmissions and metrics evaluating network performance. For example, the VDoS-LRS dataset is rich in network data, including detailed metrics like packet sizes, throughput, and packet transmission times, which are key to understanding vehicular communication networks.
- Sensor and Device Data: Sensor data involve readings from various devices installed in or around vehicles, such as cameras, GPS, and radar. This data category is increasingly important for environment sensing and enabling advanced vehicle functionalities. The DeepSense 6G dataset, with its inclusion of GPS data and radar measurements, is a prime example, offering crucial data for the development and enhancement of advanced driver-assistance systems.
- Driver and Behavioral Data: This category provides insights into driver behavior and actions. It includes data on driver identification, as well as actions like turns or signal usage. For instance, the Warrigal dataset includes data such as instantaneous speed and turn information, reflecting various aspects of driver behavior in a detailed manner.
- Safety and Incident Reporting: Safety metrics and incident data are key to understanding the safety aspects of vehicular travel. This includes information on vehicle safety systems and reports of road incidents. The PeMS dataset, for example, contributes significantly in this area with its incident reports that provide information on road accidents or construction work affecting traffic flow.
- Advanced Data Processing and Analysis: Features under this category include noise-added data for robustness testing and advanced signal processing for sophisticated data analysis. The VeReMi and the VeReMi extension exemplify this category with features like ‘‘pos_noise" and $‘‘spd_{n}oise"$, which introduce artificial noise to positional and speed data, thereby enabling the testing of algorithms under varied and challenging conditions.

Table 7 provides a summary of the most commonly used features in inter-vehicular datasets.

### 7.2. Intra-Vehicular Features

**Figure 4:**
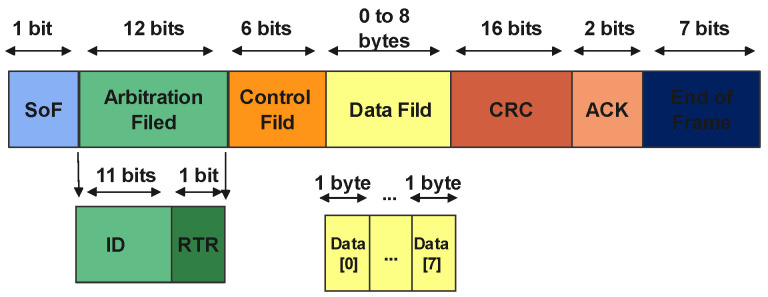
Structure of the CAN frame.

The dataset for an intra-vehicular network is composed of several key features, each playing a critical role in capturing and conveying the intricate details of the vehicle’s internal communications and functions. Most intra-vehicular datasets comprise key features that are components of the CAN bus frame (see Figure 4). In Ref. [67], the authors investigated CAN data for development purposes, defining a new CAN dataset to introduce an open-access resource for automotive intrusion detection. Each feature plays a crucial role in capturing and conveying the intricate details of the vehicle’s internal communications and functions. These features include the following.

- Timestamp: This feature records the exact moment when a data point is logged, providing a temporal reference that is crucial for tracking the sequence and timing of events within the vehicle’s network.
- CAN ID: This identifier is required for distinguishing the myriad types of messages and commands transmitted across the vehicle’s network. It is essential to categorize and understand the different communications occurring within the vehicle’s systems.
- Data Length Code (DLC): Representing the length of the data field in a message, this feature is key to understanding the size and format of the transmitted data, giving insight into the complexity and nature of the messages.
- DATA[0] to DATA[7]: These data bytes form the core content of the CAN message. Each byte, up to eight in total, can carry crucial information ranging from sensor outputs, like speed or temperature readings, to control commands directed at various vehicle electronic components.

## 8. A Guide to Selecting Suitable Vehicular Datasets Depending on the Research Requirements

Selecting a suitable dataset for vehicular security research may take time due to many factors. In this section, we intend to help researchers by giving them a set of recommendations and scenarios that help in choosing the suitable dataset based on the context parameters. Therefore, we concentrate on many key metrics to guide researchers of intelligent vehicles in making informed decisions.

**Figure 5:**
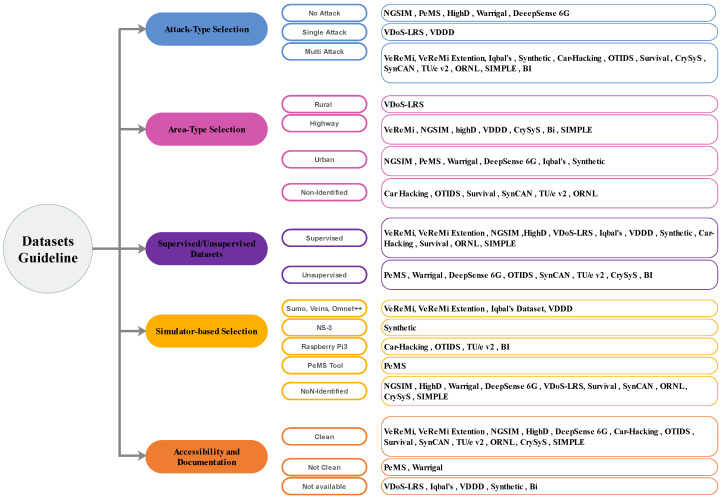
Associative scheme of the selection guideline with the suitable datasets for each case.

Firstly, confirming that the dataset is relevant to the current study issue or problem is critical. This means that the dataset should include specific information about the real-life situation the researchers are seeking to simulate or examine. We categorize these selection modules and classify them into five classes (See Figure 5). These datasets are detailed in Table 8. They are described as follows.

- Attack-Type Selection: Researchers should carefully consider the metrics and objectives of their study when choosing a dataset for research on IoV attacks. This includes the types of attacks involved in the dataset and whether to use a single-attack or multi-attack dataset. A single attack dataset would focus on a particular attack type, such as DoS (e.g., VDoS-LRS Dataset, VDDD Dataset). This dataset helps analyze a specific countermeasure’s efficacy or assess a certain attack’s impact on a network. On the other hand, a multi-attack dataset includes representations of different types of attacks and is effective for assessing the network’s resilience to a range of attack scenarios. These datasets include VeReMi/VeReMi Extention Datasets, Iqbal’s Dataset, Synthetic Dataset, Car-Hacking Dataset, OTIDS dataset, Survival Dataset, CrySyS Dataset, SynCAN Dataset, TU/e v2 Dataset, ORNL Dataset, SIMPLE Dataset, and BI Dataset. The research objectives and the threat environment of vehicle networks should guide the selection of attack types. For instance, researchers can choose to concentrate on attacks that exploit vulnerabilities in certain communication protocols or on attacks that target the network’s physical components. In addition, they can take into account the severity and frequency of attacks in the real world and the possible effects on the safety and privacy of connected vehicles. There are other datasets without attacks like GSIM Dataset, PeMS Dataset, HighD Dataset, Warrigal Dataset, and DeeepSense 6G Dataset.
- Area-Type Selection: IoV researchers looking for security datasets should consider the type of area that the dataset is focused on. The area type can be divided into three main categories: rural, highway, and urban. First, rural areas are less densely populated than urban areas, making them more challenging to secure. In this type, the researchers consider the usage of the VDoS-LRS Dataset. However, researchers can develop and test security mechanisms that are robust enough to ensure IoV security in various situations by simulating attacks in these areas. Accidents, collisions, and congestion are the primary concerns for highway areas. Therefore, the dataset (e.g., VeReMi Dataset, NGSIM Dataset, highD Dataset, VDDD Dataset, CrySyS Dataset, Bi Dataset, and SIMPLE Dataset), including information on traffic flow, accidents, and congestion levels, maybe the best fit for researchers interested in highway areas. Finally, the focus is on security threats like unauthorized vehicle access in urban areas. It is easier for an attacker to gain vehicle access in densely populated urban areas. For this type, there are multiple datasets used, like NGSIM Dataset, PeMS Dataset, Warrigal Dataset, DeepSense 6G Dataset, Iqbal’s Dataset, and Synthetic Dataset. In addition, the density of people and vehicles can make it easier for hackers to intercept and steal sensitive data, such as location and personal information.
- Simulator-based Selection: The simulators used to create a dataset can be an essential consideration when choosing a dataset for IoV security research. Different simulators may generate datasets with diverse properties, such as the size of the network, the number, and the types of attacks that can be simulated. There are various datasets whose usage adapts to these simulators. For example, sumo is adapted with VeReMi/VeReMi extension Dataset, Iqbal’s Dataset, and VD Dataset. In the same context, using a Raspberry Pi3, multiple datasets might be used, such as the Car-Hacking Dataset, the OTIDS Dataset, the TU/e v2 Dataset, and the BI Dataset. Therefore, while choosing a dataset, examining which simulator was used to produce the dataset and whether it suits the research objectives is essential. For example, to design and test a test security method using the Veins simulator, it can be beneficial to select a dataset that was generated using Veins as this can guarantee that the dataset is compatible with the simulator and that the results are more relevant to the research.
- Supervised/Unsupervised Datasets: Another essential factor when selecting an IoV security dataset is whether the dataset is supervised or unsupervised. A supervised dataset (including VeReMi /VeRemi Extension Dataset, NGSIM Dataset, and High Dataset) labels known attacks and patterns, allowing ML models to be trained to identify and react to these attacks. This dataset helps develop security mechanisms that require previous knowledge of attack patterns. On the other hand, unsupervised datasets (such as PeMS Dataset, Warrigal Dataset, and DeepSense 6G Dataset, among others) are without pre-labeled attack patterns and rely on ML models to recognize anomalies or deviations from normal behavior. These datasets are used to develop and test security models that detect previously unknown attacks.
- Accessibility and Documentation: Accessible and well-documented IoV security datasets are essential for researchers who want to perform experiments and assess various security techniques and approaches. A dataset that is accessible should be simple to obtain and use, with clear instructions on how to access and download it. A well-documented dataset should also give researchers specific information on the data, such as the types of attacks included, the vehicle types, and the communication protocols. A well-documented dataset should also contain information on the limitations of the dataset, including any assumptions or simplifications made during the development of the dataset. This information can assist researchers in understanding the dataset and its possible applications and limits. In addition, a well-documented dataset should provide instructions on adequately utilizing the dataset, such as preprocessing the data, choosing good evaluation metrics, and comparing results across studies.

**Figure 6:**
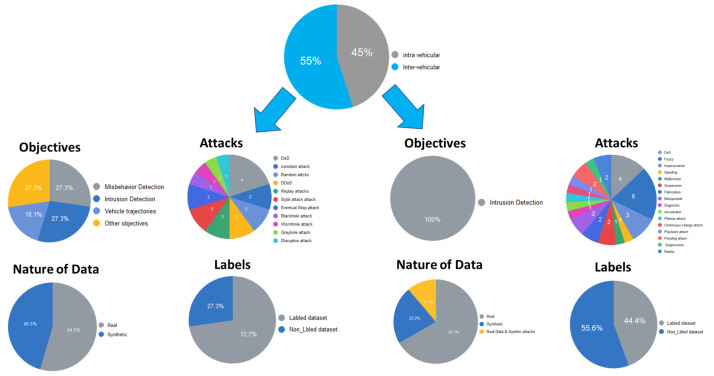
A global analysis illustration of the existing studied datasets of IoV security.

Finally, to give a global representation of the existing dataset studied in our paper, we enumerate the advantages and limitations of the existing datasets in Table 9 and Table 10. Additionally, we also present a complete graphical summary of these datasets in Figure 6, giving a visual representation that enhances understanding and analysis.

## 9. Conclusions

Vehicular network security datasets provide information used for attack detection and security enhancement. Therefore, researchers use these datasets to develop more effective security measures and protocols and ensure the safety and reliability of vehicular networks. This paper presents a comprehensive survey of vehicular network security datasets. In our survey, we have described first a brief background about the vehicular network environments, sensors, and security issues. Then, we proposed a new taxonomy on existing vehicular network security datasets. Our taxonomy is classified into six main categories: objective, data nature, data format, cyber threats, communication types, and communication protocols. The taxonomy introduced in this manuscript is a key contribution that offers a structured and systematic approach to categorizing and evaluating vehicular network security datasets. Each category in our taxonomy has been carefully selected to address specific dimensions of vehicular network security. In addition, we explore key features of both intra-vehicular and inter-vehicular datasets, which are important for enhancing vehicular network security and functionality. Inter-vehicular datasets offer insights into vehicle dynamics, traffic patterns, and communication data essential for traffic management and safety analysis. Intra-vehicular datasets focus on the vehicle’s internal operations through the CAN bus system, documenting timestamps, CAN IDs, Data Length Codes, and data bytes from internal sensors and controllers. Finally, we provided a global analysis of the different datasets to guide vehicular network researchers in selecting the best dataset according to their needs. This guide assists vehicular network researchers in selecting the dataset that best aligns with their research objectives, ensuring that they can effectively address the specific challenges of vehicular network security.

## Figures and Tables

**Table 1:** Table of Nomenclatures.

Abbreviation	Meaning
AI	Artificial Intelligence
ADUS	Archived Data User Service
BSM	Basic Safety Messages
CALM	Continuous Air-interface, Long and Medium range
CAN	Controller Area Network
CSV	comma-separated values
C-V2X	Cellular vehicle-to-everything
DL	Deep Learning
D2D	Device-to-Device
DSRC	Dedicated Short-Range Communication
ECUs	electronic control units
EDRs	Event Data Recorders
E/E	electrical and electronic
FCC	Federal Communications Commission
GN	GeoNetworking
HCRL	Human Centered Robotics Laboratory
IoT	Internet of Things
IoV	Internet of Vehicle
IETF	Internet Engineering Task Force
IDS	Intrusion Detection Systems
ITS	Intelligent Transportation Systems
LTE	Long-Term Evolution
LIN	Local Interconnect Network
ML	Machine Learning
OBU	onboard unit
OEMs	original equipment manufacturers
PeMS	Performance Measurement System
PSSCH	Physical Sidelink Shared Channels
QoS	Quality of Service
RCP	resource command processor
RSU	roadside units
SB-SPS	Sensing-Based Semi-Persistent Scheduling
TBs	Transport Blocks
TTC	Time to Collision
THW	Time Headway
V2B	Vehicle-to-Barrier
V2D	Vehicle-to-Driver
V2G	Vehicle-to-Grid
V2H	Vehicle-to-Home
V2I	Vehicle-to-Infrastructure
V2P	Vehicle-to-Pedestrian
V2R	Vehicle-to-Roadside
V2S	Vehicle-to-Sensors
V2V	Vehicle-to-Vehicle
V2X	Vehicle-to-Everything
VANETs	Vehicular ad-hoc networks
VCN	Vehicular Computing Networks

**Table 2:** Comparative table of related works.

Study	Inter-Vehicular Datasets											Intra-Vehicular Datasets									Taxonomy	Recommnedation
	VeReMi [19]	VeReMi Extension [20]	NGSIM [21]	PeMS [22]	highD [23]	Warrigal [24]	DeepSense 6G [25]	VDoS-LRS [26]	Iqbal’s Dataset [27]	VDDD [28]	Synthetic [29]	Car-Hacking [30]	OTIDS [31]	Survival [32]	SynCAN [33]	TU/e v2[34]	ORNL [35]	CrySyS [36]	SIMPLE [37]	Bi [38]		
[14]	X		X					X		X	X	X	X	X	X	X	X					
[15]	X	X	X					X	X	X	X	X	X	X	X	X	X	X	X	X		
[16]	X	X						X	X	X		X	X	X	X	X	X	X	X			
[17]									X	X		X	X	X	X	X	X	X	X			
[18]					X			X		X	X	X	X	X	X		X	X				
Our Work	X	X	X	X	X	X	X	X	X	X	X	X	X	X	X	X	X	X	X	X	X	X

**Table 3:** Classification of intra-vehicular network communication protocols.

Network Speed	Bandwidth	Topology	Max Supported Nodes	Advantages	Limitations
CAN	25 Kbps–1 Mbps	Star, Ring, Linear bus	30	High reliability, low cost	Limited bandwidth, vulnerable to attacks
LIN	25 Kbps–1 Mbps	Liner bus	16	Bus Low cost, low power	Limited data rate and distance
FlexRay	Up to 10 Mbps	Star, Linear bus, hybrid	22	High reliability, high bandwidth	Higher cost, limited interoperability
Ethernet	Up to 100 Mbps	Star, Linear bus	Depends on Switch ports	High bandwidth, scalable	Higher cost, high power consumption
MOST	Up to 150 Mbps	Ring	64	High bandwidth, low latency	Limited distance, higher cost
LVDS	Up to 3 Gbps	Point-to-point, multipoint	2–3	High data rate, low power, noise immunity	Shorter cable length, limited to simpler topologies

**Table 4:** Vehicular environment attacks.

Attack Type	Target Layer	Primary Goal	Method of Attack	Potential Impact	Detection Difficulty	Attacker Sophistication Level	Attack Range	Persistence	Scalability	Required Access
DoS	Multiple	Disrupt service	Overloading requests	System failure	Medium	Low to Medium	Variable	Temporary	High	Network Access
Jamming	Physical	Block communication	Frequency interference	Data transmission blocked	Hard	Low to Medium	Local	Temporary	Low	Proximity
Injection	Multiple	Insert false data	Unauthorized data input	Misinformation	Medium	Medium	Variable	Varies	Medium	Network Access
Tampering	Physical	Alter system/data	Physical interference	Malfunctions	Easy	Low	Proximity	Persistent	Low	Physical Access
Eavesdropping	Physical	Intercept data	Passive data capture	Information theft	Hard	Low	Local	Varies	Low	Proximity
DDoS	Multiple	Disrupt service	Distributed sources	Service unavailability	Medium	Medium to High	Wide	Temporary	High	Remote Access
Following	Network	Track movements	Message monitoring	Stalking	Easy	Low	Local	Temporary	Low	Network Access
Sybil	Network	Subvert trust	Fake identities creation	Network unreliability	Medium	Medium	Wide	Varies	High	Network Access
Spoofing	Network	Masquerade identity	Impersonation	Unauthorized access	Medium	Medium	Variable	Temporary	Medium	Network Access
MiTM	Network	Intercept/alter data	Communication interception	Data manipulation	Hard	High	Local	Temporary	Low	Network Access
Black Hole	Network	Data disruption	Data packet dropping	Communication breakdown	Medium	Medium	Local	Temporary	Low	Network Access
Sink Hole	Network	Selective disruption	Selective packet dropping	Unreliable communication	Medium	Medium	Local	Temporary	Low	Network Access
Worm Hole	Network	Data tunneling	Tunnel creation	Security bypass	Hard	High	Wide	Persistent	Medium	Network Access
Grey Hole	Network	Partial disruption	Intermittent dropping	Unreliable reliability	Medium	Medium	Local	Varies	Medium	Network Access
Phishing	Application	Steal credentials	Deceptive communication	Financial loss	Medium	Low	Wide	Temporary	High	User Interaction
Malware	Application	Disrupt/Control system	Software installation	System damage	Medium	Medium to High	Variable	Persistent	High	User Interaction
Replay	Application	Unauthorized access	Data retransmission	Security bypass	Easy	Low	Local	Temporary	Low	Network Access

**Table 5:** Inter-vehicular communication datasets comparative table.

Dataset	Ref./Year	Objective	Attacks	Nature of Data	Number of Vehicles	Format	Label	Protocol
VeReMi	[19] 2018	Misbehavior Detection	constant attacker, constant offset attacker, random attacker, random offset attacker, eventual stop attacker.	Synthetic	225	JSON	Yes	DSRC
VeReMi Extension	[20] 2020	Misbehavior Detection	DoS, DoS Random, Data Replay, Disruptive, Eventual Stop, Traffic congestion Sybil	Synthetic	2846 scenario 1, 1179 scenario 2, 7399 scenario 3	JSON	Yes	DSRC
NGSIM	[21] 2016	vehicle trajectory prediction	NA	Real	9206 Vehicles, 8860 cars, 278 Trucks	Different Formats	Yes	NA
PeMS	[22] 2002	Traffic Detection, Incident detection	NA	Real	400,000	NA	No	NA
highD	[23] 2018	Vehicle trajectories	NA	Real	110,000 Vehicles, 90,000 cars, 20,000 Trucks	CSV	Yes	NA
Warrigal	[24] 2014	road mapping, driver intent prediction, collision avoidance	NA	Real	NA	CSV	No	NA
DeepSense6G	[25] 2022	Blockage identification and prediction, object detection/classification	NA	Real	NA	Different Formats	No	NA
VDoS-LRS	[26] 2020	Intrusion Detection	DoS	Real	2	NA	Yes	IEEE 802.11 g
Iqbal’sDataset	[27]	Misbehavior Detection	Replay attack, Bogus information attack	Synthetic	5	CSV	Yes	DSRC, 5G (C-V2X)
VDDD	[28] 2021	Intrusion Detection	DDoS	Synthetic	20 Vehicles in low traffic rate, 60 vehicles in high traffic rate	different formats	Yes	Ethernet, IEEE 802.11,
Synthetic	[29] 2018	Intrusion Detection	DoS, DDoS Grayhole, Blackhole, Wormhole, Sybil	Synthetic	30 (Vehicles and Hots)	NA	Yes	WiFi, IPv4 AODV, ARP

**Table 6:** Intra-vehicular communication datasets: comparison.

Dataset	Ref./Year	Objective	Attacks	Nature of Data	Format	Labeled Data	Protocol
Car-Hacking	[30] 2018	Intrusion Detection	DoS, Fuzzy, Spoofing	Real	CSV	Yes	CAN protocol
OTIDS	[31] 2017	Intrusion Detection	DoS, Fuzzy, Impersonation	Real	CSV	No	CAN protocol
Survival	[32] 2018	Intrusion Detection	Flooding, Fuzzy, Malfunction	Real	CSV	Yes	CAN protocol
SynCAN	[33] 2019	Intrusion Detection	Suspension, Fabrication, Masquerade	synthetic	CSV	No	CAN protocol
TU/e v2	[34] 2019	Intrusion Detection	DoS, Fuzzy, Diagnostic, Replay, Suspension	synthetic	CSV	No	CAN protocol
ORNL	[35] 2020	Intrusion Detection	Masquerade, Fabrication targeted ID, Accelerator	Real	CSV	Yes	CAN protocol
CrySyS	[36] 2021	Intrusion Detection	Plateau attack, Continuous change attack, Playback attack, Suppression attack, Flooding attack	Real data and synthetic attacks	CSV	No	CAN protocol, GPS
SIMPLE	[37] 2019	Intrusion Detection	Dominant Impersonation, Complete Impersonation	Real	NA	Yes	CAN protocol
Bi	[38] 2022	Intrusion Detection	Dos, Fuzzy, Ulterior Fuzzy, Replay	Real	NA	No	CAN protocol

**Table 7:** Inter-vehicular features: a comparison.

Category	Feature	Metric Type	Description	Use Case
Vehicle Dynamics and Positioning	Lateral (X) and Longitudinal (Y) Coordinates	Coordinate Data	Position of the vehicle on the road	Traffic pattern analysis
	Instantaneous Velocity	Speed Measurement	Speed of the vehicle at a given moment	Speed regulation enforcement
	Instantaneous Acceleration	Acceleration Rate	Rate at which vehicle speed changes	Driving behavior analysis
Temporal Data	Timestamps	Time Duration	Exact date and time for data capture	Event sequencing
	Duration	Time Interval	Time elapsed for specific events or states	Congestion analysis
Vehicle Identification and Characteristics	Vehicle Identification Numbers	Identifier	Unique ID for each vehicle	Vehicle-specific analysis
	Vehicle Types	Classification	Type of vehicle (car, truck, etc.)	Traffic management
Traffic and Congestion Analysis	Traffic Volume	Count	Number of vehicles passing a point	Traffic flow analysis
	Traffic Density	Density Measurement	Vehicles per mile/kilometer	Congestion assessment
Network and Communication Data	Packet Transmission Data	Data Transmission	Information on data packets in network	Network performance analysis
Sensor and Device Data	Camera Feeds	Images or Video	Visual data from cameras	Collision avoidance
	Radar Data	Proximity Data	Detection of objects and distances	Object detection
Driver and Behavioral Data	Speed Patterns	Speed Measurement	Consistent speeding, frequent stops	Driver behavior monitoring
	Lane Change Frequency	Behavioral Data	Frequency of changing lanes	Traffic pattern analysis
Safety and Incident Reporting	Incident Reports	Descriptive Data	Reports of road incidents	Safety assessment and response
Infrastructure and Environmental Factors	Road Type and Condition	Infrastructure Data	Physical condition of the road	Infrastructure planning
	Traffic Signal Status	Operational Data	Status of traffic lights	Traffic control
Advanced processing and Analysis	Noise-Added Data	Analytical Data	Data with added noise for robustness testing	Algorithm testing

**Table 8:** Representative guideline table of all existing VN datasets.

Dataset	Attack type	Area type	Simulator	Supervised	Documentation
VeReMi	Multi-attacks	Congested highways, free-flowing traffic	Sumo, Veins, Omnet++	Supervised	Clear
VeReMi Extension	Multi-attacks	Urban	Sumo, Veins, Omnet++	Supervised	Clear
NGSIM	No attacks	Highway, Uban	-	Supervised	Clear
PeMS	No attacks	Urban Area Freeways	PeMS Tool	Unsupervised	Unclear
highD	No attacks	highways	-	Supervised	Clear
Warrigal	No attacks	Urban	-	Unsupervised	Unclear
DeepSense 6G	No attacks	Urban	-	Unsupervised	Clear
VDoS-LRS	One attack	Rural	-	supervised	Not available
Iqbal’s Dataset	Multi-attacks	Urban	Sumo, Veins, Omnet++	Supervised	Not available
VDDD	One attack	Highway	Sumo, Veins, Omnet++	Supervised	Not available
Synthetic	Multi-attacks	Urban	NS-3	Supervised	Not available
Car-Hacking	Multi-attacks	-	Raspberry Pi3	Supervised	Clear
OTIDS	Multi-attacks		Raspberry Pi and Arduino	Unsupervised	Clear
Survival	Multi-attacks	-	-	Supervised	Clear
SynCAN	Multi-attacks		-	Unsupervised	Clear
TU/e v2	Multi-attacks	-	two Arduino boards	Unsupervised	Clear
ORNL	Multi-attacks	-		Supervised	Clear
CrySyS	Multi-attacks	Highway	CAN Log Infector tool	Unsupervised	Clear
SIMPLE	Multi-attacks	Highway	TivaC microcontroller, SN65HVD230 CAN transceiver	Supervised	Clear
Bi	Multi-attacks	country roads, highways, and congested city roads	Raspberry Pi	Unsupervised	Not available

**Table 9:** Advantages and limitations of inter-vehicular environment security datasets.

Dataset	Advantages	Limitations
VeReMi	A high number of vehicles used for data collection. It takes into consideration the attacker probability.	Few simulated attacks. Synthetic data used to train IDS are unreliable and representative of IoV properties.
VeReMi Extension	The dataset includes a high number of simulated attacks. It includes physical error models.	Synthetic data used to train IDS are unreliable and representative of IoV properties.
NGSIM	High recorded Distance (500–640 m). High number of lanes (5–6 per direction).	The dataset does not include vehicular attacks. The dataset includes erroneous trajectory behavior. NGSIM data also have a few varieties.
PeMS	It integrates a wide variety of information	
highD	highD data have a wide variety. highD includes data of more than 16.5 h of recordings.	Few maneuvers detected for use in safety validation. highD dataset does not include vehicular attacks.
Warrigal	High number of data collected. The dataset contains a variety of information, including vehicle state information, communication logs, and received strength measurements for radio communications	The dataset does not include vehicular attacks.
DeepSense 6G	It is a large-scale real-world dataset comprising co-existing and synchronized multi-modal sensing and communication data. High number of scenarios (more than 40).	The dataset does not include vehicular attacks.
VDoS-LRS	This dataset was generated and labeled based on a realistic testbed. It takes into consideration different types of environments (urban, rural, and highway)	It includes a few attack scenarios. Only two vehicles are used for data collection.
Iqbal’s Dataset	The proposed scenarios mimic the real-world scenarios.	Synthetic data used to train IDS are unreliable and representative of IoV properties.
VDDD	VDDD dataset is generated based on complete traffic captured from all the nodes. The simulation scenario contains all the VANET components (RSU, vehicles and routers)	Synthetic data used to train IDS are unreliable and representative of IoV properties. The dataset does not satisfy heterogeneity and attack diversity.
Synthetic	It takes into consideration different attacks that are not considered in the other dataset (Black Hole, Gray Hole, Wormhole).	Unavailable dataset.Synthetic data used to train IDS are unreliable and representative of IoV properties.

**Table 10:** Advantages and limitations of intra-vehicular environment security datasets.

Dataset	Advantages	Limitations
Car-Hacking	The attack captures are very long and contain a large number of instances per attack. This dataset seems to be the most widely used in the CAN IDS research community.	All the attack captures contain a significant artifact of data collection that may pose a problem for researchers using this data. Ambient and attack data are in different formats.
OTIDS	It is the only open dataset that includes remote frames and responses. The fuzzing attack is unique in being the sole example of this kind of fuzzing attack in an open dataset.	The documentation on the injection message intervals needs to be clarified. The “impersonation attack” is not a real masquerade attack because the legitimate node’s message transmission is suspended.
Survival	It contains real attacks on multiple vehicles. This dataset provides evidence for the importance of the duration during which the bus is occupied by a message.	All of the attacks are basic and can be detected with a very simple frequency-based detector. only 60–90 s of data are provided per vehicle, which is likely not sufficient for robust training. the ambient data and attack data are in differently formatted CSVs, which is undesirable.
SynCAN	This is the only dataset (other than ours) that contains attacks targeting a single signal. This dataset contains the most nuanced masquerade attacks currently available.	Synthetic data are clearly an imperfect proxy for real data. Simulated attacks are inherently problematic since their effect on a vehicle cannot be verified.
TU/e v2	This dataset includes the only diagnostic protocol attack publicly available and the only suspension attack (simulated) in real CAN data. The same set of attacks is available for testing on multiple vehicles/CANs.	Attack labels are in an unstructured text file, so there is no way of programmatically reading what/when packets were injected. Most of the attacks are unrealistic.
ORNL	The published data have been obfuscated in a way that maintains the anonymity of the vehicle while preserving all important aspects of the data for an IDS.	Unlabeled data.
CrySyS	This dataset can be easily extended to add new attacks. This is the only dataset furnished with descriptions of the driver’s actions during ambient captures, which is highly valuable for training and testing an IDS.	Attacks are added in post-processing, there is no guarantee that these attacks would actually affect vehicle function. Can-Log-Infector’s implementation can cause many problems.
SIMPLE	The dataset handles both periodic and aperiodic messages.	Documentation needs to be clarified.
Bi	Provides a robust foundation for IDS under challenging conditions and comprehensive evaluation of detection capabilities.	Unavailable dataset (Private and not accessible)
